# Molecular Characterization of Viable *Legionella* spp. in Cooling Tower Water Samples by Combined Use of Ethidium Monoazide and PCR

**DOI:** 10.1264/jsme2.ME14115

**Published:** 2015-01-16

**Authors:** Hiroaki Inoue, Reiko Fujimura, Kunio Agata, Hiroyuki Ohta

**Affiliations:** 1 Tsukuba Research Laboratories, Aquas Corporation4–4 Midorigahara, Tsukuba, Ibaraki 300–2646Japan; 2 Ibaraki University College of Agriculture3–21–1 Chuou, Ami-machi, Ibaraki 300–0393Japan

**Keywords:** clone library, Ethidium monoazide (EMA), *Legionella*, quantitative polymerase chain reaction (qPCR), Viable but nonculturable (VBNC)

## Abstract

Viable *Legionella* spp. in environmental water samples were characterized phylogenetically by a clone library analysis combining the use of ethidium monoazide and quantitative PCR. To examine the diversity of *Legionella* spp., six cooling tower water samples and three bath water samples were collected and analyzed. A total of 617 clones were analyzed for their 16S rRNA gene sequences and classified into 99 operational taxonomic units (OTUs). The majority of OTUs were not clustered with currently described *Legionella* spp., suggesting the wide diversity of not-yet-cultured *Legionella* groups harbored in cooling tower water environments.

*Legionella* species are Gram-negative bacteria that are ubiquitously found in natural and man-made water systems. In aquatic environments, *Legionella* spp. can proliferate as the intracellular parasites of free-living protozoa (8, 19). The human inhalation of aerosols from *Legionella*-contaminated waters, mainly from cooling tower waters and bath waters, often results in a severe form of pneumonia called Legionnaires’ disease (legionellosis) (22). Therefore, the control of *Legionella* populations in water systems and monitoring for *Legionella* contamination are very important areas in public health microbiology.

The populations of *Legionella* spp. in environmental water samples have so far been estimated by culture-based plate counting and culture-independent molecular methods using the quantitative polymerase chain reaction (qPCR). Many attempts to grow environmental strains of *Legionella pneumophila*, the main causative agent of legionellosis, on plate media have been successful, and have provided insights into the ecology of *L. pneumophila* in natural environments (9). Regarding molecular methods, *Legionella* genus-specific (14) and *L. pneumophila* species-specific (13) PCR assays have been developed and proven to be valuable tools for investigating *Legionella* contamination in water systems. The molecular detection of *Legionella* spp. by conventional PCR methods could not previously distinguish viable bacterial cells from viable but nonculturable (VBNC) and dead cells (15, 24). However, the use of DNA-intercalating dyes such as ethidium monoazide (EMA) and propidium monoazide (PMA) before PCR was recently found to be effective for the specific amplification of DNA from *Legionella* cells maintaining membrane integrity (17). EMA and PMA can penetrate membrane-damaged cells and form covalent links with DNA, and such labeled genomic DNA within damaged cells is degraded upon exposure to visible light. The use of EMA (2–4, 6, 11, 18) and PMA (3, 20, 25) for the PCR quantification of *Legionella* has been described previously. However, to the best of our knowledge, the *Legionella* groups detected by the EMA- or PMA-treated PCR method have not yet been fully characterized.

Therefore, the aim of the present study was to determine whether the *Legionella* groups detectable by EMA-treated PCR belonged to known *Legionella* spp.. We compared the diversities of viable *Legionella* groups in six cooling tower water samples and three bath water samples by constructing clone libraries. As a result, 617 clones from *Legionella* spp. were recovered and their sequences determined from the water samples.

Water samples were collected from six different cooling towers (sample ID; CTW-A, -B, -C, -G, -H, and -I) and three different baths (BW-D, -E, and -F) between November 2012 and January 2014. Water quality control management prior to our water sampling is described in Supplementary Table S1. Samples were taken in sterile 500-mL polypropylene bottles with 0.05% (w/v) sodium thiosulfate, kept in the dark at 4°C until microbiological plating and DNA extraction, and used for these analyses within 3 d.

*Legionella* populations in water samples were enumerated according to the standard culture method (12). Briefly, water samples were subjected to centrifugation at 6,400×*g* for 30 min and the precipitate was suspended in one-100th the volume of the initial water sample. A portion of the suspension was mixed with the same volume of acid-phosphate buffer (10), and after 10 min, inoculated onto GVPC selective agar plates (Merck, Tokyo, Japan). These plates were incubated at 37°C for 8 d. Isolates of *Legionella* from the GVPC selective agar plates (50 to 100 strains per sample, except for the very low-CFU sample [four strains, CTW-G] and the sample with CFUs below the detection limit [CTW-H]) were tested by the immune serum aggregation assay (Denka Seiken, Niigata, Japan) and DNA-DNA hybridization assay (Kyokuto Pharmaceutical Industrial, Tokyo, Japan). 16S rRNA gene sequences were determined for strains that were negative for both assays, as described below.

*L. pneumophila* Phladelphia-1 (ATCC 33152) was used as the standard in qPCR assays, and cultured on a buffered charcoal yeast extract medium supplemented with 2-ketoglutarate (BCYEα) (7) at 30°C. The genomic DNA of *L. pneumophila* cells was extracted by the alkaline-boil method of Beige *et al.* (1) and purified using a NucleoSpin gDNA Clean-up kit (TaKaRa Bio, Otsu, Japan) according to the manufacturer’s instructions. The copy number of *Legionella* 16S rRNA genes was calibrated using a Cycleave PCR *Legionella* (16S rRNA) Detection Kit (TaKaRa Bio).

A Viable *Legionella* Selection Kit for PCR ver.2.0 (TaKaRa Bio) including the EMA treatment was used for the clone library construction, as described by the manufacturer. Briefly, 1 mL of the 100-fold concentrated water sample was further concentrated to a final volume of 40 μL and mixed with 10 μL of the kit reaction buffer, 2.5 μL of the kit dilution buffer, and 2.5 μL of EMA reagent. After gently mixing using a vortex mixer and incubating in the dark for 15 min at room temperature, the samples were exposed to visible light for 15 min in a LED Crosslinker 12 (TaKaRa Bio). Thereafter, DNA was extracted and purified from each sample as described above.

Populations of *Legionella* spp. and *L. pneumophila* were quantified by qPCR using the primer pairs LEG-225F (5′-AAG ATT AGC CTG CGT CCG AT-3′) and LEG-858R (5′-GTC AAC TTA TCG CGT TTG CT-3′) (14), and *Lmip*L920 (5′-GCT ACA GAC AAG GAT AAG TTG-3′) and *Lmip*R1548 (5′-GTT TTG TAT GAC TTT AAT TCA-3′) (13), respectively. PCR reaction mixtures (30 μL) contained 5 μL of template DNA, 1 μL of 10 μM forward primer, 1 μL of 10 μM reverse primer, 0.15 μL of Ex *Taq* polymerase, 2.4 μL of dNTPs, 3 μL of 10×Ex buffer (TaKaRa Bio), and 1 μL of 1,000 dilutions of SYBR Green I dye (Lonza, ME, USA) with dimethyl sulfoxide in a Thermal Cycler Dice Real Time System *II* (TaKaRa Bio). The PCR program parameters were: an initial denaturation step of 2 min at 95°C followed by 45 cycles of denaturation for 15 s at 95°C, annealing for 30 s at 65°C (LEG primer pair) or 50°C (*Lmip* primer pair), and extension for 60 s at 72°C. A melting curve analysis was performed to detect the presence of primer dimers after the final extension by increasing the temperature from 50 to 95°C in 0.5°C increments every 10 s. The calibration qPCR was performed using *L. pneumophila* DNA, and the copy numbers of *Legionella* 16S rRNA genes were quantified as described previously: PCR performance was confirmed to be reproducible at the threshold cycles (Ct) <37 (11). Furthermore, the ratios of *L. pneumophila* were calculated from the amounts of *Legionella* 16S rRNA genes and *L. pneumophila mip* genes. To construct clone libraries, PCR using primers LEG-225F and LEG-858R was carried out according to the protocol of Nishizawa *et al.* (16) to minimize PCR bias: an initial denaturation step of 2 min at 95°C followed by each threshold cycle as determined by qPCR, denaturation for 15 s at 95°C, annealing for 30 s at 65°C, and extension for 60 s at 72°C. The reaction mixture (30 μL) was composed of 5 μL of template DNA, 1 μL of 10 μM LEG-225F primer, 1 μL of 10 μM LEG-858R primer, 0.15 μL of Ex *Taq* polymerase, 2.4 μL of dNTPs, and 3 μL of 10×Ex buffer (TaKaRa Bio) in a Thermal Cycler SP (TaKaRa Bio). The PCR products were purified by using a QIAquick PCR purification kit (Qiagen, CA, USA), ligated with the vector pMD20-T using a Mighty TA-cloning kit (TaKaRa Bio), and the ligation products were used to transform *E. coli* DH5α Competent Cells (TaKaRa Bio) according to the manufacturer’s instructions. The nucleotide sequences of clones were determined with a BigDye Terminator Cycle Sequencing Ready Reaction Kit (Applied Biosystems, CA, USA) using M13 primer RV (5′-CAG GAA ACA GCT ATG ACC-3′) or M13 primer M4 (5′-GTT TTC CCA GTC ACG AC-3′) according to the manufacturer’s instructions and were read on an Applied Biosystems 3130xl genetic analyzer. Operational taxonomic units (OTUs) were defined as sequences with at least 99% similarity of all clones based on an analysis using Mothur platform software (http://www.mothur.org). The phylogenetic tree was constructed by the neighbor-joining method using MEGA5 software. Diversity indices (Chao 1, Simpson, Shannon-Wiener, and Good’s coverage) were calculated on Mothur platform software at a cut-off level of 0.01 (99% sequence identity with gaps) in the average neighbor method.

The 16S rRNA gene partial sequences were deposited in DDBJ with accession numbers AB857847 to AB858225 and AB933772 to AB934017.

Fig. 1 shows a graphical representation of the relationships between viable population densities of *Legionella* spp. in the tested water samples determined by the standard culture method (horizontal axis) and those by the EMA-qPCR method targeting the *Legionella* 16S rRNA genes (vertical axis). When *L. pneumophila* ATCC33152 was used as a positive control, the relationship between CFU 100 mL^−1^ (*x*) and the 16S rRNA gene copy numbers 100 mL^−1^ (*y*) was approximated as an equation, *y* = 0.45*x*^1.05^ (*r*^2^ = 0.996). Culturable *Legionella* counts ranged from <10 to 7.6×10^4^ CFU 100 mL^−1^ and the copy numbers of *Legionella* 16S rRNA genes from viable cells were between 6.0×10^2^ and 2.4×10^5^ 100 mL^−1^. Four (CTW-B, -C, -G, and -I) of the six cooling tower water samples contained approximately 10^5^ 100 mL^−1^ *Legionella* 16S rRNA gene copies, which was approximately 100-fold higher than that in the other samples (CTW-A and -H) and 10- to 100-fold higher than that in the bath water samples. *Legionella* viable counts for all bath water samples and the three cooling tower water samples (CTW-A, -C, and -I) were >10^3^ CFU 100 mL^−1^, which was higher than that in the other cooling tower water samples. Four sample plots (CTW-A, CTW-C, BW-D, and BW-E) were close to the positive control line, while the plots of the other cooling tower water samples (CTW-B, -G, -H, and -I) deviated markedly upward from the line, suggesting that these samples contained larger *Legionella* populations that were unable to grow under the tested culturing conditions than the culturable ones, which was also found in our recent study (11). The identification of isolates by the immunoassay and the DNA-DNA hybridization assay revealed the dominance of *L. pneumophila*, accounting for >79% of the total *Legionella* populations, except for the very low-CFU sample (only four isolates for CTW-G) and one sample that was below the detection limit (CTW-H). The sequences of the 16S rRNA genes from all four isolates from CTW-G and one from CTW-B were 100% identical to those from *Legionella* sp. LC2720 and *Legionella* sp. L-29, respectively. Approximately 20% of the isolates from BW-D were identified as *Legionella dumoffii*.

Fig. 2 shows the neighbor-joining tree based on the *Legionella* 16S rRNA gene partial sequence (616 bp) from the cooling tower and bath water samples. A total of 617 clones (cooling tower waters: 417 clones, bath waters: 200 clones) were recovered from the water samples and classified into 99 OTUs at a cut-off level of 0.01 (99% sequence identity). Good’s coverages of these libraries were 82.9% to 96.0% (cooling tower waters) and 96.9% to 98.6% (bath waters). The most abundant OTU, represented by clone ctw-A-9 (137 clones, 22% of all clones), clustered with the *L. pneumophila* group (Fig. 2). The dominance of *L. pneumophila* in BW-E and BW-F was confirmed by the clone library analysis, accounting for 99% and 51% of clones, respectively. In the other bath water sample (BW-D), *L. pneumophila* was also the main member (34%) of the clone library. In contrast, the percentage of *L. pneumophila* clones was very low in the cooling water samples: less than the detection limit for CTW-G and CTW-H and 1 to 11% for the other cooling tower water samples. The second most abundant OTU, represented by clone bw-D-15 (43 clones, 7% of all clones), was affiliated with the *L. maceachernii* cluster and accounted for 65% of the clones from BW-D and 1% of those from BW-E. The other clones that clustered with known *Legionella* spp. were *L. feeleii* (13 clones, 2% of all clones), *L. lytica* (three clones, 0.5% of all clones), and *L. dumoffii* (one clone, 0.2% of all clones).

Although the clone sequences that clustered with the *L. maceachernii* sequence were abundant in BW-D, this organism was not detected by the plate culture method. These results may be explained by either its VBNC state or a failure to outcompete *L. pneumophila* in the culture. On the other hand, *Legionella* sp. L-29 and *Legionella* sp. LC2720 were not detected by the clone libraries from CTW-B and -G, respectively. It is likely that although the plate culture method detected their very low population densities, the coverage of our clone library was too low to detect them.

Diversity indices were calculated and are summarized in Supplementary Table S2. The Chao1 values of these libraries were 10 to 67 (cooling tower water) and 2 to 7 (bath water). The Simpson (1/λ) values of these libraries were 2.08 to 13.32 (cooling tower water) and 1.03 to 2.58 (bath water). The Shannon-Wiener (*H*′) values of these libraries were 1.14 to 2.67 (cooling tower water) and 0.07 to 1.06 (bath water). All these indices suggested that the diversity of *Legionella* communities present in cooling tower water was higher than that in bath water, and may be explained by differences in water treatments. Bath water was cleaned with a higher concentration of chlorine for a shorter period of time than cooling tower water, which may have resulted in the selective survival of chlorine-resistant strains. Further studies will be needed to clarify the relationship between the diversity of *Legionella* floras and the treatment of water systems.

A number of clones (390 clones, 63% of all clones) showed less than 99% similarity to the sequences of the known culturable *Legionella* spp. strains or uncultured *Legionella* clones. Thirty clones (7 OTUs) were closely related to the uncultured *Legionella* sp. clone SEC03 (10 clones) from the cooling tower water (23), the uncultured bacterium clone SBR09C-OTUSBR10 (10 clones), the uncultured bacterium clone T0-Ps-25C-20 (21) (four clones), the uncultured *Legionella* sp. clone SEC17 (23) (two clones), the uncultured bacterium clone E9 (two clones), *Legionella* sp. FM-3-661 (one clone), and *Legionella* sp. S090 (5) (one clone).

In conclusion, our results showed that the EMA-PCR method was capable of revealing more diverse *Legionella* groups than the standard culture method and is, thus, a better tool for monitoring *Legionella* contamination in various environments.

## Supplementary Information



## Figures and Tables

**Fig. 1:**
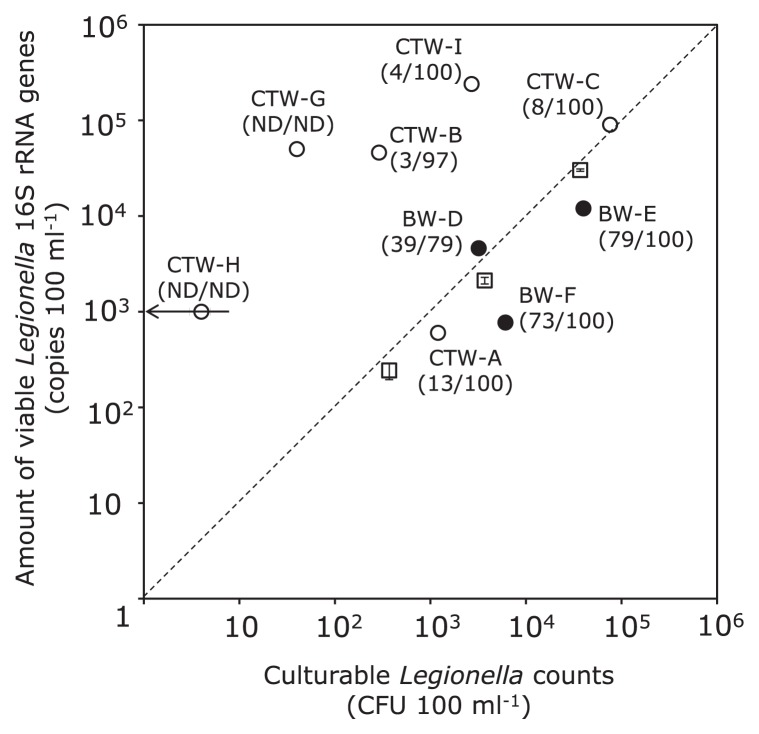
Viable population densities of *Legionella* spp. in tested cooling tower (open circles), bath (solid circles) water samples, and cell suspensions of *L. pneumophila* ATCC33152 (open squares) determined by the standard culture method (horizontal axis) and EMA-qPCR targeting 16S rRNA genes (vertical axis). CTW-A to -C and -G to -I, and BW-D to -F represent the sample ID. The number pair in parentheses under the sample ID shows the percentage of the *Legionella pneumophila* population density relative to the total *Legionella* spp. population density, which was estimated by qPCR specific for *L. pneumophila* (left figure) and the identification of isolates (right figure); ND, not detected. Arrow on the sample CTW-H symbol shows that the sample harbored <10 CFU 100 mL^−1^ of *Legionella* spp. The relationship between CFU 100 mL^−1^ (*x*) and the 16S rRNA gene copy number 100 mL^−1^ (*y*) in *L. pneumophila* ATCC33152 suspensions was approximated as a dotted straight line, *y* = 0.45*x*^1.05^ (*r*^2^ = 0.996) (duplicate determinations).

**Fig. 2:**
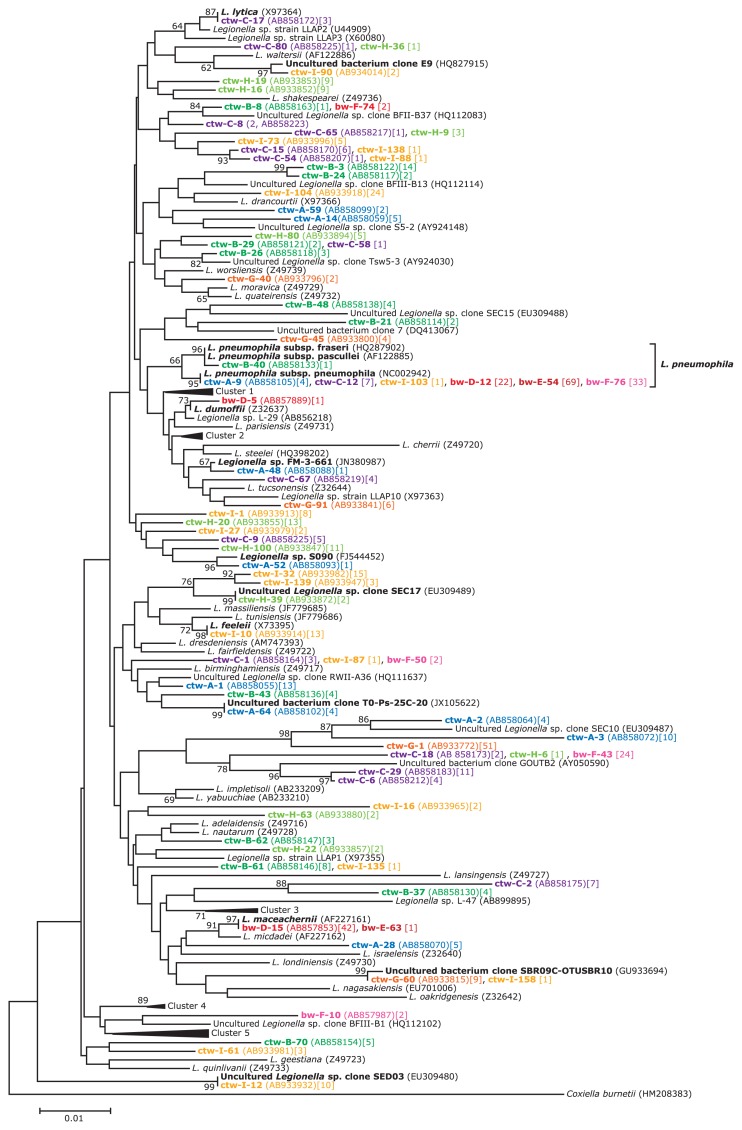
Neighbor-joining tree based on the alignment of 616-bp 16S rRNA gene sequences of 79 major representative *Legionella* clones, the *Legionella* spp. that have been described to date, and the related known uncultured *Legionella* clones. Numbers at nodes are bootstrap percentages (based on 1,000 resamplings); only values above 60% are shown. The sources of the clones were expressed in different color letters: CTW-A, blue; CTW-B, green; CTW-C, purple; CTW-G, orange; CTW-H, lime green; CTW-I, yellow; BW-D, red; BW-E, brown; BW-F, pink. After the representative clone, the accession number of the representative clone and the number of similar sequences (based on a 1% cut-off) are given in parentheses and square brackets, respectively. Cluster 1 includes *L. anisa* (Z32635), *L. bozemanii* (Z49719), *L. wadsworthii* (Z49738), *L. gormanii* (Z32639), and *L. steigerwaltii* (Z49737). Cluster 2 includes *L. sainthelensi* (Z49734), *L. santicrucis* (HF558374), *L. longbeachae* (AY444740), *L. cicinatiensis* (Z49721), and *L. gratiana* (Z49725). Cluster 3 includes *L. beliardensis* (AF122884), *L. busanensis* (AF424887), and *L. gresilensis* (AF122883). Cluster 4 includes *L. rubrilucens* (Z32643), *L. taurinensis* (DQ667196), and *L. erythra* (Z32638). Cluster 5 includes *L. brunensis* (Z32636), *L. cardiac* (JF831047), *L. hackliae* (M36028), *L. jamestownensis* (Z49726), *L. jordanis* (Z32667), and *L. spintensis* (M36030).
