# Supplementary material for: Molecular Characterization of Viable Legionella spp. in Cooling Tower Water Samples by Combined Use of Ethidium Monoazide and PCR
Source: Microbes Environ. 2015 Jan 16;30(1):108–12. doi: 10.1264/jsme2.ME14115 (PMC4356457; doi:10.1264/jsme2.ME14115)
Supplement: Supplementary file 1 [file 30_108_s1.pdf]

Supplementary tables

Table S1. Water quality control management before water sampling

Sample ID	Sampling site	Treatment condition	Concentration of biocide	Sampling date
Cooling tower waters				
CTW-A	a building in Niigata	no treatment	none	November 6, 2012
CTW-B	a plant in Tokyo	continuous supply of CMI [a]	<0.1 mg L^{-1} (CMI)	February 7, 2013
CTW-C	a factory in Yamanashi	intermittent supply [b] of CMI	<0.1 mg L^{-1} (CMI)	February 14, 2013
CTW-G	a factory in Saitama	continuous supply of CMI	<0.1 mg L^{-1} (CMI)	December 3, 2013
CTW-H	a building in Kanagawa	continuous supply of CMI	<0.1 mg L^{-1} (CMI)	January 17, 2014
CTW-I	a factory in Fukuoka	continuous supply of stabilized chlorine	2.0 mg L^{-1} (total chlorine)	November 29, 2013
Bath waters				
BW-D	a sports club in Kanagawa	continuous supply of free chlorine	1.0 mg L^{-1} (free chlorine)	March 21, 2013
BW-E	a hotel in Miyagi	continuous supply of free chlorine	no data (free chlorine)	April 23, 2013
BW-F	a sports club in Osaka	continuous supply of free chlorine	0.5 mg L^{-1} (free chlorine)	May 15, 2013

[a] 5-Chloro-2-methyl-4-isothiazolin-3-one

[b] The water treatment chemicals including CMI were batch fed as necessary.

Table S2. Diversity indices of *Legionella* communities

Sample	No. of clones analyzed	No. of OTUs obtained	Chao1	Simpson (1/λ)	Shannon-Wiener (*H*')	Good's coverage (%)
CTW-A	51	11	15	7.87	2.16	92.2
CTW-B	58	17	23	10.60	2.53	87.9
CTW-C	62	16	28	13.32	2.63	87.1
CTW-G	75	8	10	2.08	1.14	96.0
CTW-H	66	19	30	9.71	2.45	84.8
CTW-I	105	29	67	9.91	2.67	82.9
BW-D	65	3	3	1.90	0.71	98.5
BW-E	70	2	2	1.03	0.07	98.6
BW-F	65	7	7	2.58	1.16	96.9
